# The state of asthma epidemiology: an overview of systematic reviews and their quality

**DOI:** 10.1186/s13601-017-0146-y

**Published:** 2017-03-29

**Authors:** Jon Genuneit, Annina M. Seibold, Christian J. Apfelbacher, George N. Konstantinou, Jennifer J. Koplin, Stefania La Grutta, Kirsty Logan, Carsten Flohr, Michael R. Perkin, for the Task Force “Overview of Systematic Reviews in Allergy Epidemiology (OSRAE)” of the EAACI Interest Group on Epidemiology

**Affiliations:** 1 Institute of Epidemiology and Medical Biometry, Ulm University, Helmholtzstr. 22, 89081 Ulm, Germany; 2 Institute of Epidemiology and Preventive Medicine, University of Regensburg, Regensburg, Germany; 3 Department of Allergy and Clinical Immunology, 424 General Military Training Hospital, Thessaloniki, Greece; 4 Murdoch Children’s Research Institute, University of Melbourne, Melbourne, Australia; 5 Institute of Biomedicine and Molecular Immunology, National Research Council of Italy, Palermo, Italy; 6 Division of Asthma, Allergy and Lung Biology, Children’s Allergies Department, King’s College London, London, UK; 7 Unit for Population-Based Dermatology Research, St John’s Institute of Dermatology, King’s College London and Guy’s and St Thomas’ NHS Foundation, London, UK; 8 Population Health Research Institute, St George’s, University of London, London, UK

## Abstract

**Background:**

Recently, we have published an overview of systematic reviews in allergy epidemiology and identified asthma as the most commonly reviewed allergic disease. Building on this work, we aimed to investigate the quality of systematic reviews in asthma using the AMSTAR checklist and to provide a reference for future, more in-depth assessment of the extent of previous knowledge.

**Methods:**

We included all 307 systematic reviews indexed with asthma, including occupational asthma, and/or wheeze from our previous search in PubMed and EMBASE up to December 2014 for systematic reviews on epidemiological research on allergic diseases. Topics of the included systematic reviews were indexed and we applied the AMSTAR checklist for methodological quality to all. Statistical analyses include description of lower and upper bounds of AMSTAR scores and variation across publication time and topics.

**Results:**

Of 43 topics catalogued, family history, birth weight, and feeding of formula were only covered once in systematic reviews published from 2011 onwards. Overall, at least one meta-analysis was conducted for all topics except for “social determinants”, “perinatal”, “birth weight”, and “climate”. AMSTAR quality scores were significantly higher in more recently published systematic reviews, in those with meta-analysis, and in Cochrane reviews. There was evidence of variation of quality across topics even, after accounting for these characteristics. Genetic factors in asthma development were often covered by systematic reviews with some evidence of unsubstantiated updates or repetition.

**Conclusions:**

We present a comprehensive overview with an indexed database of published systematic reviews in asthma epidemiology including quality scores. We highlight some topics including active smoking and pets, which should be considered for future systematic reviews. We propose that our search strategy and database could be a basis for topic-specific overviews of systematic reviews in asthma epidemiology.

**Electronic supplementary material:**

The online version of this article (doi:10.1186/s13601-017-0146-y) contains supplementary material, which is available to authorized users.

## Background

The state of current knowledge of asthma epidemiology has been summarised in numerous narrative expert reviews including the Global Atlas of Asthma of the European Academy of Allergy and Clinical Immunology and the European Lung White Book of the European Respiratory Society [1, 2]. With an ever increasing number of systematic reviews on asthma epidemiology, systematic overviews of these systematic reviews become more and more important to keep track of the evidence, to prevent redundancy, and to provide comprehensive summaries informing decision makers. However, to date there are only two overviews of systematic reviews in asthma epidemiology and both only cover specific aspects of asthma epidemiology [3, 4].

One overview of systematic reviews, providing a meta-analysis of risk and protective factors on childhood asthma, included 42 systematic reviews published up to January 2016 [3]. Of note, this was focused on childhood, on non-genetic factors, and on systematic reviews with meta-analysis. Another overview of systematic reviews was restricted to the association of diet with asthma [4]. We are also aware of three systematic reviews which have examined the original literature rather than systematic reviews on some areas of asthma epidemiology: The first searched for articles describing risk factors for asthma incidence and the second aimed at comprehensively reviewing the original literature on selected risk and protective factors for asthma [5, 6]. The third conducted a more specific search to identify original articles on the genetic predisposition to asthma and atopy over a period of 6 years [7].

Recently, we have published a comprehensive overview on systematic reviews in allergy epidemiology which has identified a total of 307 systematic reviews covering asthma and wheeze [8]. Building on our previous work, we aimed here to investigate the systematic reviews’ quality using the AMSTAR checklist [9] and to provide a reference for future, more in-depth assessments of individual topic areas.

## Methods

The complete search strategy has been published previously [8]. In brief, we searched PubMed and EMBASE (via OVID, including conference abstracts) for systematic reviews on epidemiological research on allergic diseases. The databases were searched from their inception without restrictions, in particular with regard to language, publication period, or data on humans; the last update of the search was carried out on December 17, 2014.

Following removal of duplicates, titles and abstracts were screened for potential relevance and in that case full-text was obtained. Exclusion criteria were: (1) clear indication of lack of a systematic search (e.g. narrative reviews or meta-analyses of data from multiple study centres), (2) no human data presented (e.g. animal data, in vitro studies, simulation studies), (3) outcome definition that did not include asthma or wheeze, and (4) the investigated topic was the management of existing disease (e.g. therapeutic intervention, patient education, secondary and tertiary prevention). References of overviews of systematic reviews were scrutinised for systematic reviews and these were included if not already identified through the search strategy. All evaluation of entries and full texts was conducted by two members of the review team independently and the senior author settled cases of disagreement. The studied diseases and topics covered by the systematic reviews were indexed as previously described; here we analyse all 307 systematic reviews covering asthma, including occupational asthma, and/or wheeze [8].

Complete citations of the included reviews were extracted; we categorized the year of (print) publication into three periods: before 2006, 2006–2010, after 2010 (bands chosen with consideration of the number of articles in each period at the time of analysis). The type of systematic review, i.e. systematic review, systematic review with meta-analysis, or overview of systematic reviews, and the studied topics covered were taken from the previously published index [8]. For the outcomes and the topics, we extracted the definitions presented in the systematic reviews and any age restrictions that were applied. Data extraction was conducted independently by two members of the review team. The lead author settled any cases of disagreement.

In addition, two members of the review team independently applied the AMSTAR checklist to all relevant full-text entries [9, 10]. This is the most frequently used validated checklist to evaluate the methodological quality of systematic reviews. It has been previously noted that the wording of the AMSTAR items and instructions is a trade-off between feasibility and reliability [11]. Because of the subjective interpretation, inter-rater agreement on some AMSTAR items may be lower than on others. Rather than solving disagreement between raters and producing one averaged AMSTAR score, we deliberately instructed one rater to be more liberal and the other rater to be more conservative in applying the AMSTAR checklist. Thus, we documented an upper bound (liberal) and a lower bound (conservative) of the total AMSTAR score that may be achieved by each systematic review. Details of the liberal and conservative criteria for each AMSTAR item are shown in Additional file 1: Table S1.

For two of the systematic reviews, the extracted data were based on the abstract and those parts of the full text that could be translated [12, 13]. Full application of the AMSTAR checklist was not possible due to the limited information included in the abstracts. Therefore, these two articles were excluded from the analyses of the AMSTAR scores.

**Fig. 1:**
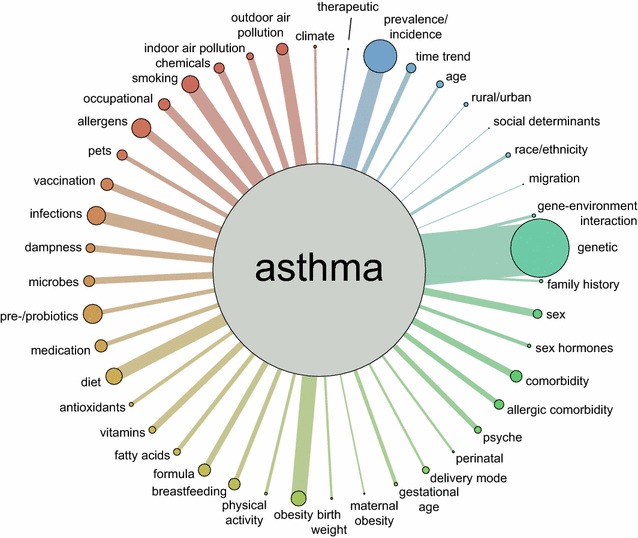
Aggregated topics indexed along with asthma. The figure is based on the aggregated indexed topics which are presented in the same clockwise order as listed in Additional file 1: Table S2. Bubble diameter is proportional to the number of systematic reviews with the respective index term (this number is based on all n = 421 systematic reviews on allergy epidemiology, also non-asthma systematic reviews (based on the aggregated disease index term “asthma”), included in our previous overview [8]). Line thickness is proportional to the number of systematic reviews indexed with the connected index terms. *Colours* are arbitrary, covering the spectrum from *blue* to *red* in clockwise order

All evaluation, data extraction, and indexing was performed using a relational database (Microsoft Access 2010^©^, Microsoft Corporation, Redmond, Washington, United States). Counts, percentages, and distributions as well as correlations and *p* values were analysed using SAS^®^ 9.3 (The SAS Institute, Cary, NC, USA). To assess differences in the systematic reviews’ quality across topics, we modelled linear regression with AMSTAR scores as the dependent variable and separate independent dummy variables per topic (each yes/no) since some systematic review were indexed with multiple topics. We report *p* values for the likelihood ratio test of the global association of topics with AMSTAR scores from these models and after further adjustment for other variables influencing the AMSTAR score. Also, we visualized the predicted mean AMSTAR scores per topic after centring all topic dummy variables and all co-variables at their respective arithmetic mean. Data visualisation in Fig. 1 was produced using Gephi^©^ (https://gephi.org/), a non-profit open-source software for network visualization and analysis created by the Gephi Consortium.

## Results

Additional file 2: Appendix S2 contains the full list of the included 307 systematic reviews, including the indexed diseases and topics as well as the AMSTAR checklist in a single spreadsheet, which can be searched and sorted. Overall, 57.0% of the systematic reviews included a meta-analysis. There were two overviews of systematic reviews, both not confined to asthma: one on second-hand smoke exposure and child health and the other on occupational safety and health interventions [14, 15].

Figure 1 depicts the aggregated topics (see Additional file 1: Table S2) indexed along with asthma or wheeze. If the line thickness matches the bubble diameter, this means that all articles indexed with that topic were indexed with asthma; if the line is thinner than the bubble diameter, the topic is also indexed solely with other allergic diseases as previously published [8]. E.g. for “obesity”, almost all systematic reviews displayed data on asthma, whereas for “pre-/probiotics”, most systematic reviews covered only other allergic diseases. Of the full list of 43 topics catalogued (see Additional file 1: Table S2), “family history”, “birth weight”, and feeding of “formula” were only covered once in systematic reviews published from 2011 onwards. The other topics were covered at least twice. Overall, at least one meta-analysis was conducted for all topics except for “social determinants”, “perinatal”, “birth weight”, and “climate”.

**Fig. 2:**
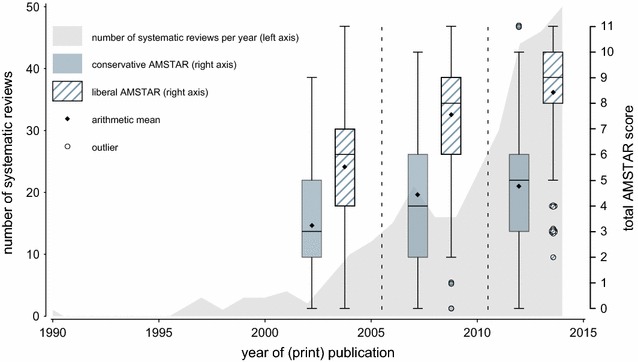
*Box plots* of AMSTAR scores over periods of publication. The *lower box margin* indicates the 1st quartile; the *upper box margin* indicates the 3rd quartile. The *vertical line* within the *box* indicates the median. *Whiskers* are drawn at the most extreme value within a distance of 1.5-times the interquartile range from the respective *box* margin. Multiple outliers at the same value are jittered

Overall, following the liberal and the conservative instructions, 63.0 and 13.1% respectively, had an AMSTAR score ≥8 which suggests good methodological quality. The AMSTAR score increased over the time period studied (Fig. 2; liberal AMSTAR: *p*_Kruskal–Wallis,2DF_ < 0.001, Spearman ρ = 0.34; conservative AMSTAR: *p*_Kruskal–Wallis,2DF_ < 0.001, Spearman ρ = 0.21). This trend happened against the background of an increasing number of systematic reviews per year over time (Fig. 2). Also, the proportion of systematic reviews with meta-analysis increased over time up to 65.5% in the most recent period from 2011 to 2014 ($p_{{\chi^{2} }} < 0.001$). In the same years, 76.3 and 11.8% had high (AMSTAR ≥8) and 5.3 and 27.2% had low (AMSTAR <4) methodological quality following the liberal and the conservative instructions, respectively. Systematic reviews with meta-analysis had a 3.0- and 2.0-points higher liberal and conservative AMSTAR score, respectively, compared to systematic reviews without meta-analysis (data not shown).

**Fig. 3:**
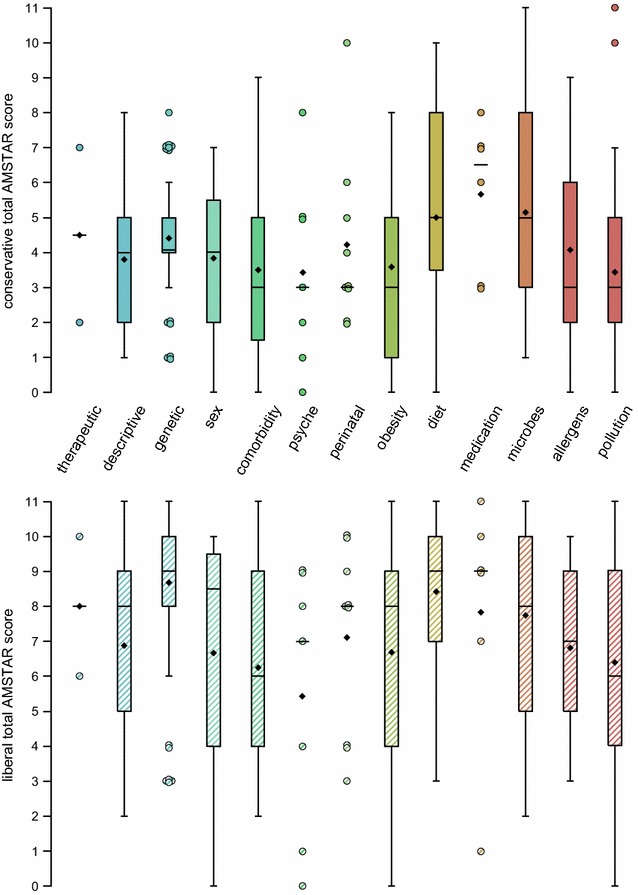
*Box plots* of conservative (*top*, solid) and liberal (*bottom*, hatched) total AMSTAR scores over indexed topics. Topics are coloured from *blue* to *red* corresponding to their underlying more detailed indices displayed in Fig. 1. Instead of *box plots*, data points are displayed for topics with less than 10 contributing systematic reviews. For description of *box plots* see legend to Fig. 2

The AMSTAR score also differed across aggregated topics (Fig. 3; Additional file 1: Figure S1; for information on aggregation of topics see Additional file 1: Table S2). This difference was not explained by effects of publication period or methodology (Additional file 1: Figure S1). Further adjustment for Cochrane versus non-Cochrane reviews resulted in decreasing average AMSTAR scores for topics with Cochrane reviews (2 for “allergens”, 7 for “diet”, 4 for “microbes”; data not shown). The global test for association of topics with AMSTAR scores in these fully adjusted models was *p*_LR-test_ = 0.063 and *p*_LR-test_ = 0.040 for the liberal and conservative AMSTAR score, respectively. The proportion of variance in AMSTAR scores explained by topics, measured as *r* ^2^ in the linear regression models, reduced from 15.3 and 10.4% in the crude models to 3.9 and 5.1% in the fully adjusted models for the liberal and conservative AMSTAR score, respectively. Overall, 48.4 and 36.4% of the variance were explained in the fully adjusted models for the liberal and the conservative AMSTAR, respectively.

Of the full list of 43 topics (see Additional file 1: Table S2), only “birth weight” did not include a systematic review reaching a liberal AMSTAR of at least 8 which indicates good methodological quality. Restricting to more recent systematic reviews published after 2010, “pets” was another topic in which the systematic review published in this period achieved a liberal AMSTAR below 8 as well. Using the conservative AMSTAR, the list of topics with all systematic reviews achieving scores below 8 is much longer: “therapeutic”, “prevalence/incidence”, “time trend”, “age”, “rural/urban”, “race/ethnicity”, “genetic”, “family history”, “sex”, “sex hormones”, “allergic comorbidity”, “perinatal”, “delivery mode”, “birth weight”, “obesity”, “physical activity”, “breastfeeding”, “dampness”, “pets”, “chemical”, “indoor air pollution”, “outdoor air pollution”, and “climate”.

The topic covered most often was “genetic” (n = 76) although this would have been outnumbered if all “environmental” topics were aggregated. Table 1 shows the number of systematic reviews pertaining to the respective genes that were investigated. Of note, most of these systematic reviews only included case–control studies and family studies were typically excluded or not discussed. Following mutual adjustment for the other topics, 87.8% of the systematic reviews on genetics had performed meta-analyses, a significantly higher proportion than for other topics. Several systematic reviews concentrated on single polymorphisms rather than all polymorphisms in a given gene or location.

**Table 1:** Genes along with the frequency they were covered by systematic reviews

Gene(s)	Number of systematic reviews
*IL4*	12
*IL13*	8
*ADAM33*, *CCL5*, *TNF*	7
*ADRB2*, *CD14*, *IL4R*	6
*GST* ^a^, *IL10*, *TGFB1*	4
*ACE*, *CXCL8*, *IFNG*, *IL18*, *MS4A2*, *TLR4*	3
*CTLA4*, *FLG*, *LTA*, *LTC4S*, *STAT6*	2
*CFTR*, *HLA*, *IL1B*, *IL9*, *MBL2*, *NAT2*, *PTGDR*, *SCGB1A1*, *SERPINE1*, *SFTPD*, *TLR2*	1

^a^Including *GSTT1* (n = 4), *GSTM1* (n = 3), and *GSTP1* (n = 2)

Four systematic reviews investigated gene-environment interactions: one on multiple genes and respiratory syncytial virus (RSV) infection [16], one on glutathione S-transferase genes and smoking [17], one on multiple genes and exposure to outdoor air pollution [18], and one on *CD14* and exposure to microbes [19]. In addition, there were two systematic reviews with meta-analysis of genome-wide linkage studies [20, 21] and one systematic review with meta-analysis of studies on the effects of maternal and paternal asthma [22]. A further systematic review investigated the association between migration status and asthma with the aim of providing information on genetic and environmental components of the disease risk [23].

## Discussion

We present a comprehensive overview of systematic reviews in asthma epidemiology including their methodological quality which demonstrates that there are systematic reviews for most of the topics identified as relevant in recent expert opinion pieces [1, 2]. Most topics have been covered by more than one systematic review and have also been covered since 2010. However, a substantial number of published systematic reviews fall short in methodological quality.

Compared to the only previous overview of systematic reviews across several topics in asthma epidemiology [3], we miss 9 systematic reviews identified by the other overview due to its 13 month longer search period up to January 2016. However, we also include 15 systematic reviews this overview failed to identify despite matching its inclusion criteria. Because this other overview was restricted to systematic reviews with meta-analysis, on childhood, and on non-genetic factors [3], our more comprehensive search identified 256 additional articles. Inevitably, such a comprehensive effort is outdated upon publication. Our previously published overview [8] suggests that about 70 systematic reviews on allergy epidemiology are published per year from 2014 on, of which about 75% are on asthma. Nonetheless and while most of the topics we identified were discussed in the previous two overviews [3, 4], our to date most extensive overview of systematic reviews on asthma epidemiology enables us to make some additions:

First, we identified 19 systematic reviews on the effects of smoking on asthma, principally covering environmental tobacco smoke exposure which is discussed in the previous overview [3]. In addition, we identified 6 systematic reviews on active smoking: one on asthma in women [24], one on gene-environment interaction with Glutathione-S-Transferase genes [17], one on marijuana smoking [25], one on the effects of smoke-free legislation [26], one evidence-based guideline on occupational asthma [27], and one on risk and protective factors [5]. The latter was published in 2004 and is the only one with a focus on the main effects of active smoking in a general population, suggesting an update is warranted.

Second, we identified several systematic reviews on the effects of specific infections other than RSV infection which is the only infection discussed in the previous overview [3]. A total of four systematic reviews on *Helicobacter pylori* infection were included, one of poor quality [28]. The other three all conducted their search up to April to July 2012 [29–31]. However, one included only five studies and found no association [29]. The other two had 12 studies in common, including the aforementioned five studies and found weaker evidence for an inverse association between *H. pylori* infection and asthma [30, 31]. This example documents how differing search and inclusion criteria may affect the overall interpretation of the assembled body of evidence. Our previous overview of systematic reviews in allergy epidemiology includes a discussion towards this issue [8].

Third, topics not covered by the previous overview include genetics (by methodology, n = 76 systematic reviews), effects of physical activity or sedentary behaviour (n = 4), pet exposure (n = 6), and formula rather than breastfeeding (n = 8, including updates). Our primary purpose was not to discuss these systematic reviews in depth but to index them and provide quality scores for future reference. Still, our results indicate that specifically birth weight was covered by systematic reviews with low methodological quality. Here, the authors of the other overview [3] identified a further systematic review [32] published after our search period which achieved an AMSTAR score of 9 in their evaluation which potentially closes this gap. Moreover, the second topic with only low quality systematic reviews published after 2010 was “pets” which was not covered by the other overview and for which a high-quality update may be warranted. In particular our conservative AMSTAR scores and our more comprehensive list may help to guide selection of further topics which may require new or updated systematic reviews. Whether an overview of systematic reviews for a specific topic or an update of an existing systematic review is warranted will also depend on scientific interest and closer investigation of the body of evidence covered by each individual systematic review.

Fourth, in our overview of systematic reviews on genetic factors implicated in asthma development, we were able to demonstrate that evidence from family-based designs has largely been ignored, even though methodology to statistically combine this evidence with that from other study designs has been suggested and applied elsewhere [33]. Moreover, as we previously discussed [8], the nature of the search for articles covering genetics may make these more amenable to quick and efficient systematic review. Furthermore, the underlying original articles have very homogenous definitions of the exposure variable (i.e. the genetic trait) and often report odds ratios which facilitates meta-analysis. For example, the effects of *IL4* polymorphisms have been investigated in 12 systematic reviews published from 2008 on with four of them in 2013 and three of them in 2014. Four of these 12 reviews included only a single polymorphism within the gene. While there may be scientific rationale to concentrate on specific polymorphisms (e.g. due to considerations of biology or those of linkage disequilibrium), it may be more appropriate to summarize evidence at the gene level. Of note, our list of genes is not a comprehensive list of genetic factors implicated in asthma development but a reflection of which of these have been investigated by systematic reviews. There are many examples of large-scale meta-analyses on genetic factors (and also other factors as we discuss in our previous publication [8]) which we did not include and which may provide high-quality evidence.

Operational definitions of asthma have previously been shown to be heterogeneous [34, 35]. In systematic reviews on asthma, its definition in the underlying original articles needs to be extracted, displayed, and discussed. This was done in a substantial number of the systematic reviews included in our overview (data not shown). Additionally, atopic and non-atopic asthma were separated in some systematic reviews. Here, specific focus should be devoted to the definition of the comparison or reference groups in the original articles, as these definitions may dramatically influence resulting associations [36]. We advocate that future systematic reviews continue to take a holistic approach with regard to specifying asthma definitions as an inclusion criterion and that they evaluate and discuss potential heterogeneity of the asthma definitions used.

While the methodological quality of the systematic reviews has been generally increasing over the past decades, there were still up to 27% with low quality scores among those published after 2010. This may of course be a high estimate due to conservative application of the AMSTAR checklist and due to inclusion of a few articles in which the authors described a systematic search but did not aim at writing a systematic review. We have deliberately applied the AMSTAR checklist twice with more liberal and with more conservative instructions rather than averaging two replicate sets to produce a range of AMSTAR scores for each systematic review. The quality score from a consensus procedure would be likely to lie within this range. Indeed, for the 33 systematic reviews included in both our list and the only previous overview supplying AMSTAR scores [3], our conservative AMSTAR was consistently lower and our liberal AMSTAR was mostly equal or higher.

The AMSTAR checklist has been previously shown to yield higher scores for Cochrane reviews, if a meta-analysis was conducted, and for more recently published systematic reviews [10, 37], all of which is reflected in our analyses. While these characteristics explained a larger portion of the overall variance in AMSTAR scores than our indexed aggregated topics, we could still detect some consistent differences across topics for both our AMSTAR definitions. The AMSTAR has also been shown to be associated with the number of pages of a systematic review [10] and the journal’s impact factor [37], but we refrained from evaluating these as we deemed them of lower importance to our aims. The AMSTAR interval we provide may guide future updates of existing systematic reviews in particular for those topics for which high quality systematic reviews are lacking as discussed above.

In conclusion, we present a comprehensive overview and an indexed database of published systematic reviews in asthma epidemiology including quality scores. We highlight some topics and issues which we believe should be considered in future systematic reviews. We propose that our results could be a basis for topic-specific overviews of systematic reviews in asthma epidemiology.

## Additional files

**Additional file 1: Tables S1.** AMSTAR checklist with “liberal” and “conservative” instructions. **Table S2.** Topic index terms and their aggregation. **Figure S1.** Profile lines of adjusted mean liberal and conservative AMSTAR scores across topics.

**Additional file 2.** Full indexed list of the included systematic reviews including AMSTAR scores.

## Abbreviations

AMSTAR: a measurement tool to assess systematic reviews
*H. pylori*: *Helicobacter pylori*
IL: interleukin
RSV: respiratory syncytial virus
