# Supplementary material for: The state of asthma epidemiology: an overview of systematic reviews and their quality
Source: Clin Transl Allergy. 2017 Mar 29;7:12. doi: 10.1186/s13601-017-0146-y (PMC5387188; doi:10.1186/s13601-017-0146-y)
Supplement: Supplementary file 1 — Additional file 1: Tables S1. AMSTAR checklist with “liberal” and “conservative” instructions. Table S2. Topic index terms and their aggregation. Figure S1. Profile lines of adjusted mean liberal and conservative AMSTAR scores across topics. [file 13601_2017_146_MOESM1_ESM.pdf]

Appendix S1

Table S1: AMSTAR checklist with “liberal” and “conservative” instructions.

AMSTAR item	liberal instruction	conservative instruction
**1. Was an 'a priori' design provided?**<br>The research question and inclusion criteria should be established before the conduct of the review.<br><br>*Note: Need to refer to a protocol, ethics approval, or pre-determined/a priori published research objectives to score a “yes.”*	sufficient if pre-determined/*a priori* objectives and methods were mentioned	only if an ethics approval was cited or a protocol or objectives and methods were published prior to the systematic review
**2. Was there duplicate study selection and data extraction?**<br>There should be at least two independent data extractors and a consensus procedure for disagreements should be in place.<br><br>*Note: 2 people do study selection, 2 people do data extraction, consensus process or one person checks the other’s work.*	sufficient if some selection or extraction was done by 2 people	only if all selection (including initial title/abstract screening) and extraction was done by 2 people
**3. Was a comprehensive literature search performed?**<br>At least two electronic sources should be searched. The report must include years and databases used (e.g., Central, EMBASE, and MEDLINE). Key words and/or MESH terms must be stated and where feasible the search strategy should be provided. All searches should be supplemented by consulting current contents, reviews, textbooks, specialized registers, or experts in the particular field of study, and by reviewing the references in the studies found.<br><br>*Note: If at least 2 sources + one supplementary strategy used, select “yes” (Cochrane register/Central counts as 2 sources; a grey literature search counts as supplementary).*	sufficient if 2 databases were searched	as originally described

AMSTAR item	liberal instruction	conservative instruction
**4. Was the status of publication (i.e. grey literature) used as an inclusion criterion?**<br>The authors should state that they searched for reports regardless of their publication type. The authors should state whether or not they excluded any reports (from the systematic review), based on their publication status, language etc.<br><br>*Note: If review indicates that there was a search for "grey literature" or "unpublished literature," indicate "yes." SINGLE database, dissertations, conference proceedings, and trial registries are all considered grey for this purpose. If searching a source that contains both grey and non-grey, must specify that they were searching for grey/unpublished lit.*	as originally described	as originally described
**5. Was a list of studies (included and excluded) provided?**<br>A list of included and excluded studies should be provided.<br><br>*Note: Acceptable if the excluded studies are referenced. If there is an electronic link to the list but the link is dead, select "no."*	sufficient if some excluded studies were referenced in an exemplary manner or if number of excluded articles per exclusion criterion were given	only if excluded studies were referenced completely for at least some exclusion criteria
**6. Were the characteristics of the included studies provided?**<br>In an aggregated form such as a table, data from the original studies should be provided on the participants, interventions and outcomes. The ranges of characteristics in all the studies analyzed e.g., age, race, sex, relevant socioeconomic data, disease status, duration, severity, or other diseases should be reported.<br><br>*Note: Acceptable if not in table format as long as they are described as above.*	as originally described	as originally described

AMSTAR item	liberal instruction	conservative instruction
**7. Was the scientific quality of the included studies assessed and documented?**<br>'A priori' methods of assessment should be provided (e.g., for effectiveness studies if the author(s) chose to include only randomized, double-blind, placebo controlled studies, or allocation concealment as inclusion criteria); for other types of studies alternative items will be relevant.<br><br>*Note: Can include use of a quality scoring tool or checklist, e.g., Jadad scale, risk of bias, sensitivity analysis, etc., or a description of quality items, with some kind of result for EACH study ("low" or "high" is fine, as long as it is clear which studies scored "low" and which scored "high"; a summary score/range for all studies is not acceptable).*	sufficient if the quality of the included studies was assessed but not displayed for each study	only if quality was assessed by a dedicated scale or restrictions or extracted items were defined for the purpose of quality assessment and if the results of quality assessment were displayed for each study
**8. Was the scientific quality of the included studies used appropriately in formulating conclusions?**<br>The results of the methodological rigor and scientific quality should be considered in the analysis and the conclusions of the review, and explicitly stated in formulating recommendations.<br><br>*Note: Might say something such as "the results should be interpreted with caution due to poor quality of included studies." Cannot score "yes" for this question if scored "no" for question 7.*	as originally described	as originally described

AMSTAR item	liberal instruction	conservative instruction
**9. Were the methods used to combine the findings of studies appropriate?**<br>For the pooled results, a test should be done to ensure the studies were combinable, to assess their homogeneity (i.e., Chi-squared test for homogeneity, I2). If heterogeneity exists a random effects model should be used and/or the clinical appropriateness of combining should be taken into consideration (i.e., is it sensible to combine?).<br><br>*Note: Indicate "yes" if they mention or describe heterogeneity, i.e., if they explain that they cannot pool because of heterogeneity/variability between interventions.*	as originally described	as originally described
**10. Was the likelihood of publication bias assessed?**<br>An assessment of publication bias should include a combination of graphical aids (e.g., funnel plot, other available tests) and/or statistical tests (e.g., Egger regression test, Hedges-Olken).<br><br>*Note: If no test values or funnel plot included, score "no". Score "yes" if mentions that publication bias could not be assessed because there were fewer than 10 included studies.*	as originally described	as originally described
**11. Was the conflict of interest included?**<br>Potential sources of support should be clearly acknowledged in both the systematic review and the included studies.<br><br>*Note: To get a "yes," must indicate source of funding or support for the systematic review AND for each of the included studies.*	sufficient if conflict of interest or source of funding was stated for the systematic review	only if (i) conflict of interest or source of funding was stated for the systematic review and (ii) conflict of interest or source of funding was stated within the systematic review for each included study<br><br>the latter criterion was already satisfied if the risk of selective reporting was judged (e.g. in Cochrane reviews)

Table S2: Topic index terms and their aggregation.

full	aggregated [1]	highly aggregated [1,2]
therapeutic intervention [3]	therapeutic	therapeutic
prevalence/incidence	prevalence/incidence	descriptive
time trend in prevalence/incidence	time trend	
age	age	
rural/urban	rural/urban	
social determinants [4]	social determinants	
race/ethnicity	race/ethnicity	
migration	migration	genetic
gene-environment interaction	gene-environment interaction	
genetic	genetic	
family history	family history	
sex	sex	sex
gender		
hormonal/puberty	sex hormones	
comorbidity [5]	comorbidity	comorbidity
endometriosis [5]		
autoimmunity [5]		
allergy [5]	allergic comorbidity	
allergic comorbidity [5]		
cytokine [5]		
psychological/psychosocial	psyche	psyche
assisted reproductive technology	perinatal	perinatal
perinatal factors		
c-section	delivery mode	
delivery mode		
gestational age	gestational age	
maternal body weight	maternal obesity	obesity
birth weight	birth weight	
obesity	obesity	
physical activity	physical activity	
breastfeeding	breastfeeding	diet
formula	formula	
fatty acids	fatty acids	
vitamins	vitamins	
antioxidants	antioxidants	
diet	diet	
medication [6]	medication	medication
pre-/probiotics	pre-/probiotics	microbes
microbes	microbes	
farming		
birth order [7]		
dampness/molds	dampness	
infections	infections	
vaccination	vaccination	
pets	pets	allergens
allergens	allergens	
occupational exposure	occupational	

smoking	smoking	pollution
chemicals	chemicals	
indoor air pollution	indoor air pollution	
outdoor air pollution	outdoor air pollution	
climate	climate	

[1] The terms are ordered from top to bottom starting with more general, descriptive concepts, going into more intrinsic topics (genetics, sex, comorbidity), and then into more environmental exposures from perinatal conditions via factors linked to obesity and diet to microbes, allergens and pollution.

[2] A second aggregation (as per companion paper(8)) was defined to allow for meaningful visualisation along with the systematic review's AMSTAR quality scores (see Figure 3 and Figure S1).

[3] Systematic reviews which present data on immunotherapy preventing the onset of subsequent allergic disease (n=2).

[4] Systematic review on area-based and individual measures of social disadvantage (n=1).

[5] Systematic reviews of articles on comorbidities were included as potential risk factors for allergic disease if they displayed data suggestive of the comorbid disease existing prior to the onset of the allergic disease.

[6] This was indexed if effects of medications for conditions other than the allergic diseases, e.g. antibiotics or paracetamol, were investigated.

[7] Systematic review on the effect of birth order (n=1); other systematic reviews dealing with proxies of the "hygiene hypothesis" were mostly indexed with "infections" and/or "microbes" depending on their content.

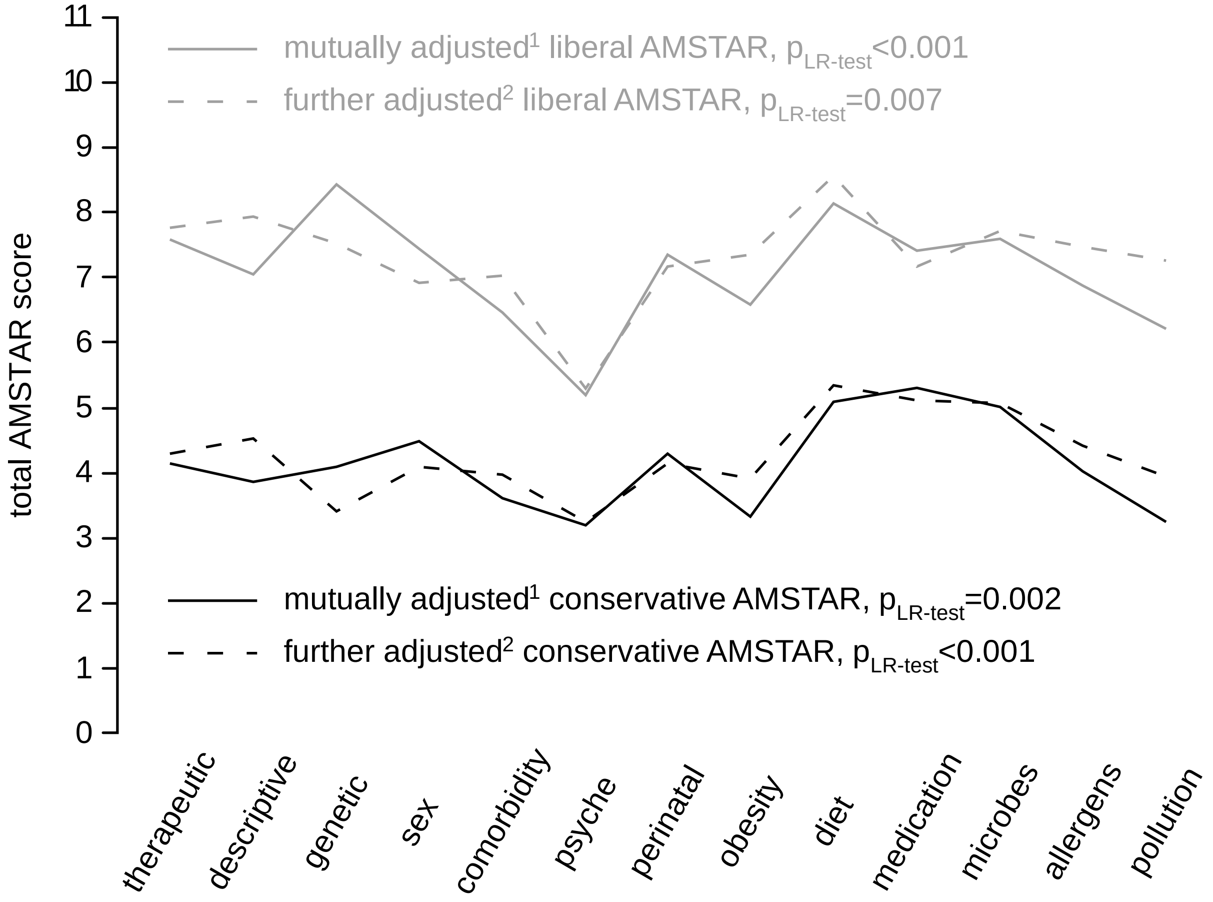

Figure S1: Profile lines of adjusted mean liberal and conservative AMSTAR scores across topics.

The profile lines connect the adjusted mean AMSTAR scores (crude means displayed by solid black rhombes in Figure 3) to facilitate identification of variation across topics and of effects of adjustment.

[1] Topics are mutually adjusted to account for systematic reviews indexed with multiple topics.

[2] Topics are mutually adjusted and further adjusted for publication period (categorically, ≤ 2005, 2006 - 2010, ≥ 2011 (reference)) and methodology (systematic review with vs. without meta-analysis (reference))
